# DGAT1 Inhibitor Suppresses Prostate Tumor Growth and Migration by Regulating Intracellular Lipids and Non-Centrosomal MTOC Protein GM130

**DOI:** 10.1038/s41598-019-39537-z

**Published:** 2019-02-28

**Authors:** Francesca Nardi, Omar E. Franco, Philip Fitchev, Alejandro Morales, Renee E. Vickman, Simon W. Hayward, Susan E. Crawford

**Affiliations:** Department of Surgery, NorthShore University Research Institute, Affiliate of University of Chicago Pritzker School of Medicine, Evanston, IL 60201 United States

## Abstract

Acyl-CoA:diacylglycerol acyltransferase I (DGAT1) is a key enzyme in lipogenesis which is increased in metabolically active cells to meet nutrient requirements. DGAT1 has been recognized as an anti-obesity target; however, its role in the tumor microenvironment remains unclear. We postulated that, in prostate cancer (PCa) cells, augmented lipogenesis and growth are due to increased DGAT1 expression leading to microtubule-organizing center (MTOC) amplification. Thus, therapeutic targeting of DGAT1 potentially has tumor suppressive activity. We tested whether blocking DGAT1 in PCa cells altered MTOC and lipid signaling. Western blot and immunofluorescence were performed for MTOC and triglyceride mediators. Treatment with a DGAT1 inhibitor was evaluated. We found a stepwise increase in DGAT1 protein levels when comparing normal prostate epithelial cells to PCa cells, LNCaP and PC-3. Lipid droplets, MTOCs, and microtubule-regulating proteins were reduced in tumor cells treated with a DGAT1 inhibitor. Depletion of the non-centrosomal MTOC protein GM130 reduced PCa cell proliferation and migration. Inhibition of DGAT1 reduced tumor growth both *in vitro* and *in vivo*, and a negative feedback loop was discovered between DGAT1, PEDF, and GM130. These data identify DGAT1 as a promising new target for suppressing PCa growth by regulating GM130, MTOC number and disrupting microtubule integrity.

## Introduction

Obesity is known to increase the risk of tumor progression in several cancers^1^. Although lipid metabolism is better characterized in adipocytes, recent studies demonstrate that cancer cells have an exceptional capacity to uptake fatty acids (FAs) and store them in lipid droplets (LDs), which, in turn, can be used to sustain proliferation and migration^2,3^. However, the factors modulating intratumoral lipid content and ectopic storage in non-fat storing cells are still not fully understood. LDs are dynamic cytoplasmic organelles which act as sites for lipid storage, membrane synthesis, and intracellular trafficking. These organelles are formed by a core of neutral lipids containing triacylglycerols (TAGs) and cholesterol esters (CEs) surrounded by a phospholipid monolayer with proteins either embedded in this monolayer or attached to its surface^4,5^. TAGs within LDs may represent a renewable source of FAs for cancer cells; although, the mechanisms by which TAG storage is regulated to provide a survival benefit to cancer cells are unclear. As cancer cells take up an increasing amount of FAs, the number and size of LDs increase^6^. The enzymes involved in the synthesis of TAGs, such as Acyl-CoA:diacylglycerol acyltransferase 1 (DGAT1)^7^, contribute to the increase of size and number of LDs, whereas lipolysis mediators such as, adipose triglyceride lipase (ATGL)^8^ and pigment epithelium-derived factor (PEDF)^9–11^, metabolize TAGs and help to maintain a lower net level of intracellular lipid stores.

DGAT1 is one of the major enzymes in TAG biosynthesis which specifically catalyzes the final step of lipogenesis converting diacylglycerol and acyl-CoA to TAG^12^. In both humans and mice, DGAT1 is highly expressed in metabolically active and fat storing tissues responsible for the synthesis of TAGs such as intestine, adipose tissue, liver, and mammary gland^13,14^. Less is known about DGAT1 expression and signaling partners in other organ systems. DGAT1 knockout mice are phenotypically lean and resistant to diet-induced obesity and fatty liver disease. They also exhibit increased insulin and leptin sensitivity and increased energy expenditure or reduced tissue TAG concentration^15–19^. Pharmacological DGAT1 inhibition studies in animal models have consistently shown a reduction in diet-induced obesity^20^ and in plasma total cholesterol concentrations^21^. These data led to the testing of inhibitors of DGAT1 in human clinical trials for the treatment of obesity and metabolic syndrome^22^, since the drugs prevent both TAG synthesis and LD formation^23^. The potential anti-tumor activity of DGAT1 inhibitors in cancer has not been explored.

In our recent study, we found that stromal cells, specifically cancer-associated fibroblasts, undergo metabolic re-programming by activating a new signaling communication between microtubule-organizing centers (MTOCs) and LDs^24^. Whether this lipid-MTOC axis is important in regulating aggressive tumor growth is not known. The best studied MTOC is the centrosome, which is mainly involved in the bipolar spindle formation during mitosis in order to ensure proper cell cycle progression^25^. Recently, there has been more interest in characterizing another group of MTOCs known as non-centrosomal MTOCs (ncMTOCs), which can vary depending on the cell type. Emerging studies have better characterized the structure and function of these ncMTOCs and revealed they have different functions from centrosomes. For example, cellular differentiation is one bioactivity that has been linked to activation of ncMTOCs which is relevant to tumor aggressiveness^26^. It has also been reported that other organelles such as mitochondria or the Golgi apparatus can convert to functional ncMTOCs through modulation of various proteins^27,28^. Among these, GM130 represents the peripheral Golgi protein regulating cell polarization and migration by affecting centrosome and microtubule (MT) organization at the Golgi^29,30^. GM130 depletion reduces the centrosome’s ability to nucleate MTs^31^, and also prevents the formation of Golgi-nucleated MTs which are important in the cell polarization, migration and intracellular trafficking^32^. The formation of Golgi-derived MTs depends on MT-stabilizing CLIP-associated proteins (CLASPs), which selectively coat Golgi-derived MTs and, thus, making them structurally and dynamically dissimilar from the centrosomal-derived MTs. Thus, the Golgi-derived MTs are unique and characterized by a specific origin, orientation, and protein composition^33^. Golgi-associated CLASPs have been shown to be important in several tumor relevant biological processes such as intracellular trafficking, cellular motility and polarization^34^.

In the present study, we show that DGAT1 is overexpressed in prostate cancer (PCa) cells compared to normal prostate epithelium, and that the inhibition of this lipogenic enzyme can reduce not only LD density, but also the ncMTOC number and MT stability. The blockade of DGAT1 affected important signaling proteins associated with ncMTOCs and reduced expression levels of γ-tubulin, GM130, and CLASP2 in the more aggressive PCa cells. Moreover, inhibition of DGAT1 resulted in the suppression of proliferation, migration and invasion in PCa cells *in vitro* and reduced growth *in vivo*. Depletion of the ncMTOC protein GM130 reduced tumor cell proliferation and migration supporting a new mechanism for anti-DGAT1 bioactivities in cancer. Taken together, these data suggest that lipogenic enzyme DGAT1 is responsible for growth of lipid-laden PCa cells, and inhibition of DGAT1 effectively reduces both amplified MTOCs and intracellular lipid content. This study highlights DGAT1 as a promising multifunctional target to suppress growth and progression in prostate cancer.

## Results

### TAG-related proteins are dysregulated and promote intracytoplasmic lipid accumulation in prostate cancer cells

**Figure 1:**
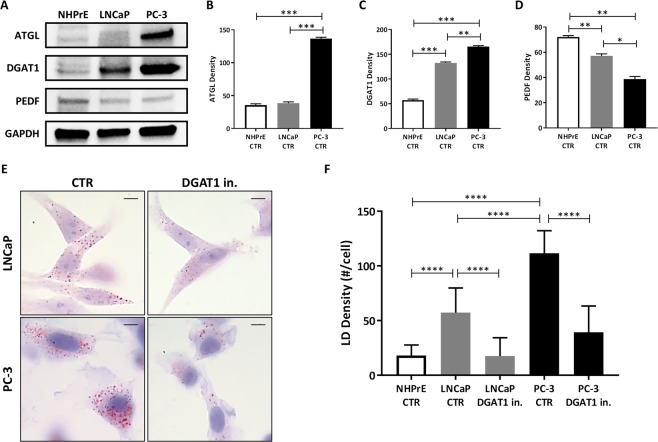
TAG-related proteins are dysregulated and suppression of DGAT1 decrease lipid content in prostate cancer cells. (**A**) ATGL (56 kD), DGAT1 (55 kD), and PEDF (50 kD) levels were analyzed by western blot in NHPrE, LNCaP and PC-3 cells. (**B**) ATGL, (**C**) DGAT1, and (**D**) PEDF densities were normalized by GAPDH in NHPrE, LNCaP and PC-3 cells. (**E**) LNCaP and PC-3 cells were treated with 1 µM DGAT1 inhibitor for 24 h, and, after fixation, LDs were analyzed with Oil-Red-O which stains neutral lipids in red. Size bar: 10 µm. (**F**) The total number of LDs per single cell in LNCaP (grey) and PC-3 cells (black) was counted (n = 50). For the western blot analysis, each protein has been detected by sequential staining and stripping using the same membrane. Data are presented as mean ± SEM. Student’s unpaired t test. *P < 0.05, **P < 0.01, ***P < 0.001, ****P < 0.0001.

Proteins that regulate TAG metabolism are located both on the surface of the LDs and in the cytoplasm and regulate intracellular lipid metabolism by promoting lipolysis (ATGL and PEDF)^8,10^ or lipogenesis (DGAT1)^7^. Expression levels of these three TAG-related proteins were assessed in PCa cells, LNCaP and PC-3, versus normal human prostate epithelium (NHPrE), using western blot analysis (Fig. 1A). When compared to NHPrE, PC-3 cells showed a 3–4 fold higher levels of both ATGL (PC-3 vs NHPrE: 136.6 ± 1.6 vs 35.6 ± 1.6; P < 0.001) (Fig. 1B) and DGAT1 (PC-3 vs NHPrE: 165.6 ± 1.5 vs 57.5 ± 1.6; P < 0.001) (Fig. 1C), indicating that in prostate cancer cells both lipolysis and lipogenesis are highly active. However, DGAT1 expression in the tumors appeared to be higher than ATGL, thereby, leading to a net gain (lipogenesis > lipolysis) in intracellular lipid content consistently observed in the cancer cells. In contrast, less aggressive prostate cancer cells, LNCaP, appeared less metabolically active in the TAG pathway compared to PC-3 cells, showing a lower expression of both ATGL (LNCaP vs PC-3: 38.5 ± 1.5 vs 136.6 ± 1.6; P < 0.001) and DGAT1 (LNCaP vs PC-3: 132.6 ± 1.5 vs 165.6 ± 1.5; P < 0.01) proteins. However, LNCaP cells still expressed higher levels of DGAT1 protein when compared to NHPrE (LNCaP vs NHPrE: 132.6 ± 1.5 vs 57.5 ± 1.6; P < 0.001) (Fig. 1C).

Unlike ATGL and DGAT1, PEDF protein, which, in addition to its role in promoting lipolysis by binding to ATGL, has also anti-tumor activity, was downregulated in both cancer cell lines when compared to NHPrE (LNCaP vs NHPrE: 57.2 ± 1.2 vs 72.3 ± 0.8; P < 0.01; PC-3 vs NHPrE: 38.6 ± 1.6 vs 72.3 ± 0.8; P < 0.01) (Fig. 1D). These results suggest that dysregulation of lipid metabolism in PCa cells involves changes in the expression of ATGL, DGAT1, and PEDF, and excessive DGAT1 could contribute to defective lipid flux and result in a net gain of intracellular lipid content.

### DGAT1 inhibitor reduces the density of LDs in prostate cancer cells

To determine whether changes in the TAG pathway, specifically the levels of DGAT1, correlate with aberrant lipid storage in PCa cells, we quantified the number of baseline LDs in LNCaP and PC-3 cells and tested if LD density changed under lipogenesis suppression by a DGAT1 inhibitor treatment. The cells were stained with Oil-Red-O which selectively stains the neutral lipids, constituting the core of LDs (Fig. 1E). Under baseline conditions, both cancer cell lines revealed a higher mean of intracellular LDs when compared to the NHPrE (LNCaP vs NHPrE: 57.3 ± 4.5 vs 18.0 ± 1.9; P < 0.0001; PC-3 vs NHPrE: 111.6 ± 5.1 vs 18.0 ± 1.9; P < 0.0001), with more aggressive PC-3 cells showing a significantly higher mean of LD density when compared to lower aggressive LNCaP (PC-3 vs LNCaP: 111.6 ± 5.1 vs 57.3 ± 4.5; P < 0.0001) (Fig. 1F). To assess whether the lipid-rich phenotype in PCa cells could be suppressed, a blockade of lipogenesis by a 24 h treatment with a DGAT1 inhibitor was tested. This treatment regimen drastically decreased the mean number of LDs by >3 fold in LNCaP (LNCaP DGAT1 in. vs LNCaP CTR: 17.5 ± 4.3 vs 57.3 ± 4.5; P < 0.0001) and by >2.5 fold in PC-3 cells (PC-3 DGAT1 in. vs PC-3 CTR: 39.4 ± 5.5 vs 111.6 ± 5.1; P < 0.0001) when compared to the baseline (Fig. 1F). After DGAT1 inhibitor treatment, the mean of LD density in PC-3 cells was still higher than LNCaP cells (PC-3 DGAT1 in. vs LNCaP DGAT1 in.: 39.4 ± 5.5 vs 17.5 ± 4.3; P < 0.01).

### DGAT1 inhibition reduces cell proliferation, migration, and invasion in prostate cancer cells

**Figure 2:**
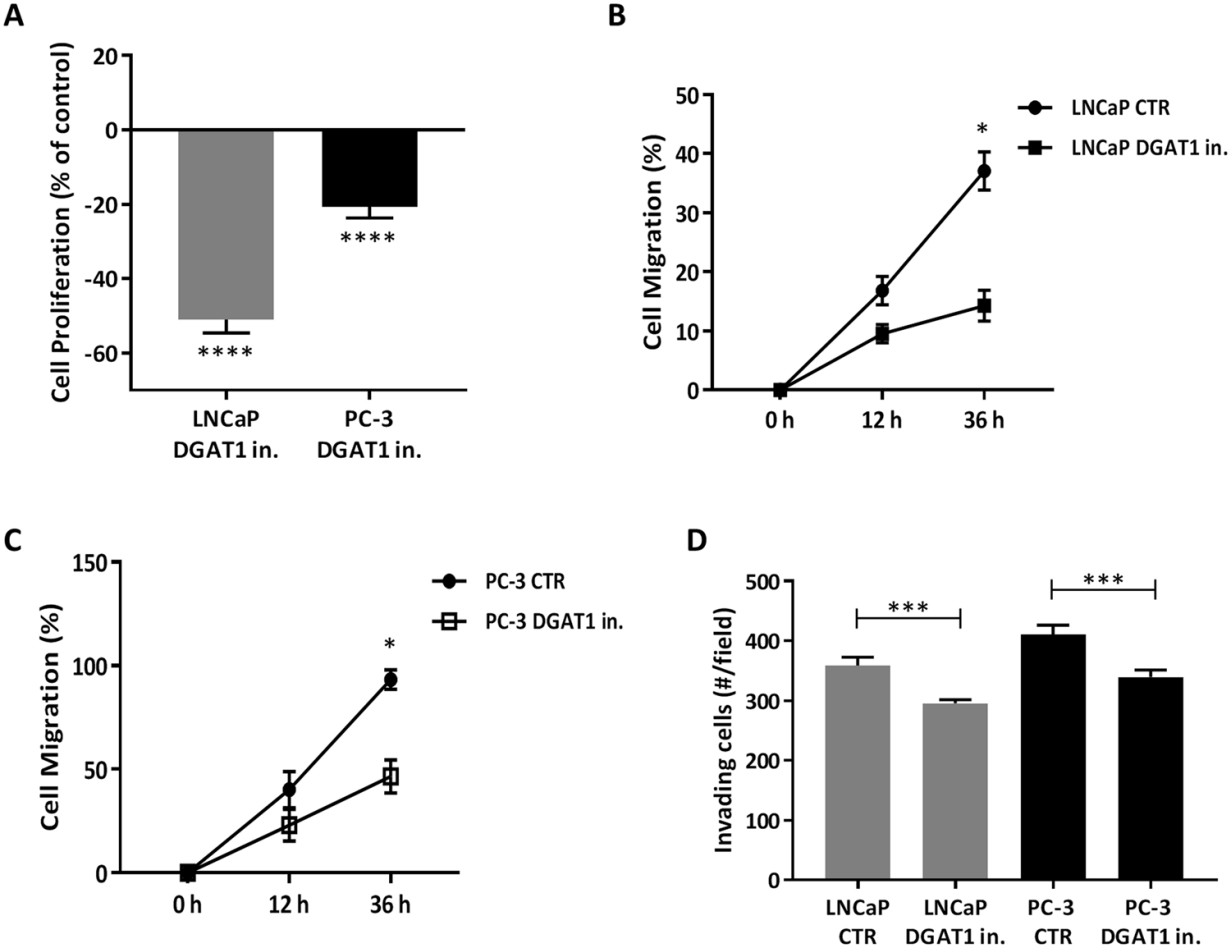
DGAT1 inhibitor reduces cell proliferation and migration in prostate cancer cells. LNCaP and PC-3 cells were treated with 1 µM DGAT1 inhibitor for 24 h. (**A**) The proliferation rate was evaluated by the percentage of cells positive for PCNA staining/total cells (n = 50). The migration rate was evaluated by the percentage of (**B**) LNCaP cells and (**C**) PC-3 cells able to move into the scratched area at the times of 12 and 36 h. (**D**) Cell invasion was analyzed at the time of 24 h. Data are presented as mean ± SEM. Student’s unpaired t test. *P < 0.05, ***P < 0.001, ****P < 0.0001.

Cancer cells generally proliferate at a higher rate than benign cells and, in some instances, this is due, in part, to the ability to access resident nutrient sources^35^. Given our data showing a high level of stored lipids in both LNCaP and PC-3 cells at baseline, it was not clear whether modulating intracellular lipid content could directly alter cellular proliferation, migration, and invasion. To assess this, both PCa cells, LNCaP and PC-3, were treated for 24 h with a DGAT1 inhibitor. Cell proliferation was then analyzed based on the percentage of proliferating cell nuclear antigen (PCNA) staining positive cells at baseline and compared after the treatment with a DGAT1 inhibitor (Fig. 2A). Our results showed that the treatment with a DGAT1 inhibitor reduced the proliferation rate by nearly 50% in LNCaP cells and 20% in PC-3 cells when compared to the respective controls (LNCaP DGAT1 in. vs LNCaP CTR: P < 0.0001; PC-3 DGAT1 in. vs PC-3 CTR: P < 0.0001) (Fig. 2A). To determine whether DGAT1 was a common enzyme utilized by other tumor cell types, we used the same DGAT1 inhibitor treatment regimen and found a similar effect in reducing the proliferative rate in both pancreatic cancer cells (PANC-1) (PANC-1 DGAT1 in. vs PANC-1 CTR: 0.2 ± 0.0 vs 1.0 ± 0.0; P < 0.0001) (see Supplementary Fig. S1) and liver cancer cells (Hepa-1c1c7) (Hepa-1c1c7 DGAT1 in. vs Hepa-1c1c7 CTR: 0.2 ± 0.0 vs 0.5 ± 0.0; P < 0.0001) (see Supplementary Fig. S1). These results suggest that the higher proliferative baseline capacity of cancer cells could be due to ready access to its robust supply of stored lipid, and this lipid depot has the potential to rapidly renewed due to the elevated level of intracellular lipogenesis enzyme DGAT1.

Cell migration and invasion are important properties for cancer cells to acquire since this allows them to invade neighboring tissue and gain access to vascular structures to facilitate metastasis. Cell migration was analyzed in PCa cells based on their ability to migrate in specific intervals of time. The analysis showed that, compared to the baseline, the treatment with a DGAT1 inhibitor significantly reduced the migration in LNCaP cells (LNCaP DGAT1 in. vs LNCaP CTR: 14.3 ± 2.6 vs 37.1 ± 3.2; P < 0.05) and in PC-3 cells (PC-3 DGAT1 in. vs PC-3 CTR: 46.6 ± 7.9 vs 93.3 ± 4.7; P < 0.05) at the time of 36 h (Fig. 2B,C). The treatment with a DGAT1 inhibitor was also able to reduce the invasive capacity of both LNCaP cells (LNCaP DGAT1 in. vs LNCaP CTR: 295.5 ± 3.1 vs 359.0 ± 6.7; P < 0.001) and PC-3 cells (PC-3 DGAT1 in. vs PC-3 CTR: 339.3 ± 6.0 vs 410.7 ± 9.0; P < 0.001) when compared to the baseline (Fig. 2D).

### Blockade of lipogenesis by a DGAT1 inhibitor reduces ncMTOC number in prostate cancer cells

**Figure 3:**
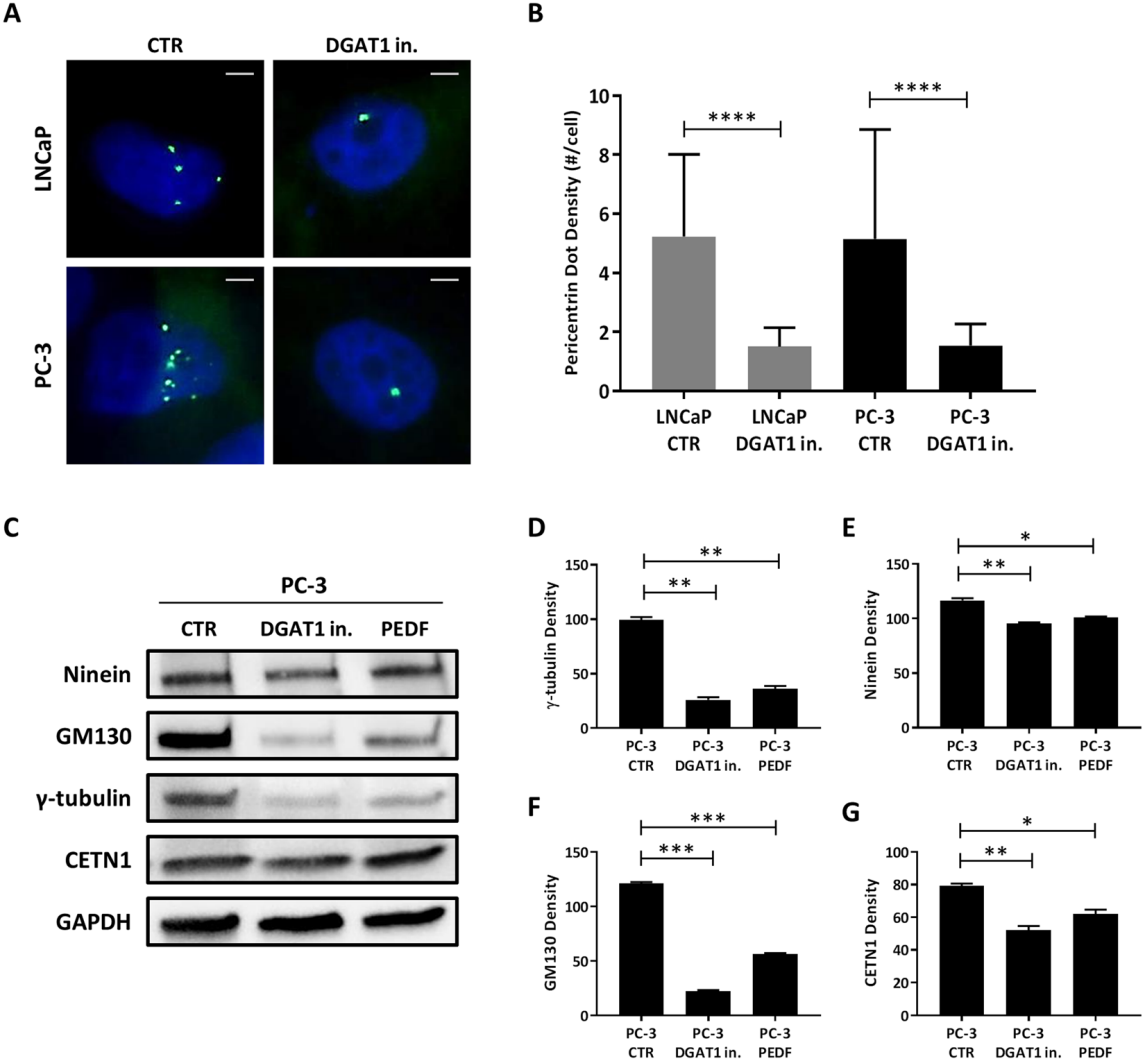
DGAT1 inhibition decreases ncMTOC-related proteins in prostate cancer cells. (**A**) LNCaP and PC-3 cells were treated with 1 µM DGAT1 inhibitor for 24 h, and, after fixation, the cells were stained with mouse anti-pericentrin antibody (green) and DAPI (blue) to visualize the MTOCs and nucleus, respectively. Size bar: 10 µm. (**B**) The total number of pericentrin-positive MTOCs was counted per single cell (n = 50). (**C**) γ-tubulin (48 kD), ninein (243 kD), GM130 (140 kD), and CETN1 (20 kD) levels were analyzed by western blot in PC-3 control (CTR) and treated with 1 µM DGAT1 inhibitor or 10 nM PEDF. (**D**) γ-tubulin, (**E**) ninein, (**F**) GM130, and (**G**) centrin 1 (CETN1) densities were normalized by GAPDH. For the western blot analysis, each protein has been detected by sequential staining and stripping using the same membrane. Data are presented as mean ± SEM. Student’s unpaired t test. *P < 0.05, **P < 0.01, ***P < 0.001, ****P < 0.0001.

MTOC abnormalities and amplification have been observed in many tumors including prostate^24,36,37^, where they seem to act by disrupting astral microtubule organization, promoting aneuploidy and tumorigenesis^38^. Since PCa cells showed a lipid imbalance with elevation of both lipolysis and lipogenesis (Fig. 1A), we sought to determine whether a change in intracellular lipid content was capable of modulating the MTOC number. To assess MTOC density in LNCaP and PC-3 cells, immunofluorescent staining was performed to detect the presence of a pericentriolar matrix protein, pericentrin, which is commonly used to denote the presence of both centrosomal and non-centrosomal MTOCs^39^. As expected, at baseline, both LNCaP and PC-3 cells revealed amplification of pericentrin-positive foci located in the perinuclear region of the cells (Fig. 3A). To assess whether inhibition of lipogenesis could normalize the MTOC number in LNCaP and PC-3, the cells were treated for 24 h with a DGAT1 inhibitor. The staining revealed that, compared to the control, the treatment had significantly reduced the number of pericentrin-positive MTOCs by 71.2% in LNCaP (LNCaP DGAT1 in. vs LNCaP CTR: 1.5 ± 0.1 vs 5.2 ± 0.5; P < 0.0001) and by 71.8% in PC-3 cells (PC-3 DGAT1 in. vs PC-3 CTR: 1.5 ± 0.1 vs 5.5 ± 0.7; P < 0.0001) (Fig. 3B). DGAT1 inhibitor treatment markedly reduced the number of MTOCs to a mean of 1–2 per cell (Fig. 3A,B), while concurrently suppressing intracytoplasmic LD density by 69.5% and 64.7% to a mean of 17.5 and 39.4 LDs/cell in LNCaP and PC-3 cells, respectively (Fig. 1E,F). The ability of a DGAT1 inhibitor treatment to reduce the density of both LDs and MTOCs in PCa cells supports the concept that MTOCs have a possible biosensor function to assess intracellular lipid content. The existence of a new signaling communication system between these two organelles, LDs and ncMTOCs, adds a new dimension for testing questions linking lipid disorders such as obesity and cancer progression.

### DGAT1 inhibition decreases ncMTOC-related proteins in more aggressive prostate cancer cells

To better understand which proteins were involved in the MTOC plasticity seen in PCa cells after a DGAT1 inhibitor treatment, western blot analysis was performed in more aggressive, high DGAT1 expressing, PC-3 cells to quantify the expression levels of proteins related to non-centrosomal and/or centrosomal MTOCs (Fig. 3C). In particular, pericentriolar matrix proteins γ-tubulin and ninein are located in both centrosomal and non-centrosomal MTOCs^40^, where γ-tubulin controls the nucleation and polar orientation of MTs, while ninein has a MT anchoring function^26^. When compared to the control, the treatment with a DGAT1 inhibitor significantly reduced the levels of γ-tubulin (PC-3 DGAT1 in. vs PC-3 CTR: 25.9 ± 1.8 vs 99.3 ± 1.9; P < 0.01) (Fig. 3D) and ninein (PC-3 DGAT1 in. vs PC-3 CTR: 95.8 ± 0.6 vs 116.5 ± 1.5; P < 0.01) (Fig. 3E). Also GM130, a protein associated with ncMTOCs and, selectively, to the Golgi-like MTOCs, was tested to investigate whether a regulatory feedback loop exists between DGAT1 and GM130. This protein has been reported to regulate MT nucleation at the Golgi and centrosome organization^29^. The treatment with a DGAT1 inhibitor strongly decreased the expression levels of GM130 (PC-3 DGAT1 in. vs PC-3 CTR: 22.4 ± 0.6 vs 121.1 ± 0.9; P < 0.001) when compared to the control (Fig. 3F). To assess whether the DGAT1 inhibitor regulated centrosomal-specific MTOCs, we analyzed the expression levels of centrin 1 (CETN1) which is a centriolar protein located exclusively in centrosomes where it exerts an important role in regulating the centrosome position and segregation during the cell cycle^41^. When compared to the baseline, the treatment with a DGAT1 inhibitor decreased the levels of centrin 1 in the more aggressive PC-3 cells (PC-3 DGAT1 in. vs PC-3 CTR: 52.0 ± 1.8 vs 79.2 ± 0.9; P < 0.01) (Fig. 3G). Since in our previous study we found that PEDF, a lipolysis-mediating protein known to suppress tumor progression, was able to normalize the MTOC number in both PCa cells and prostate stromal cells by reducing pericentrin and centrin 1 proteins to a single focus per cell, we tested potential signaling mechanisms responsible for the ability of PEDF to alter MTOCs. PC-3 cells were treated with PEDF and expression levels of MTOC-related proteins were analyzed (Fig. 3C). When compared to the control, the treatment with PEDF reduced the expression levels of all the following proteins critical to MTOC integrity and function: γ-tubulin (PC-3 PEDF vs PC-3 CTR: 36.0 ± 2.0 vs 99.3 ± 1.9; P < 0.01) (Fig. 3D), ninein (PC-3 PEDF vs PC-3 CTR: 100.8 ± 0.7 vs 116.5 ± 1.5; P < 0.05) (Fig. 3E), GM130 (PC-3 PEDF vs PC-3 CTR: 56.4 ± 0.5 vs 121.1 ± 0.9; P < 0.001) (Fig. 3F), and centrin 1 (PC-3 PEDF vs PC-3 CTR: 62.1 ± 1.8 vs 79.2 ± 0.9; P < 0.05) (Fig. 3G).

### DGAT1 inhibition regulates critical MT-associated proteins and disrupts the MT network in more aggressive prostate cancer cells

**Figure 4:**
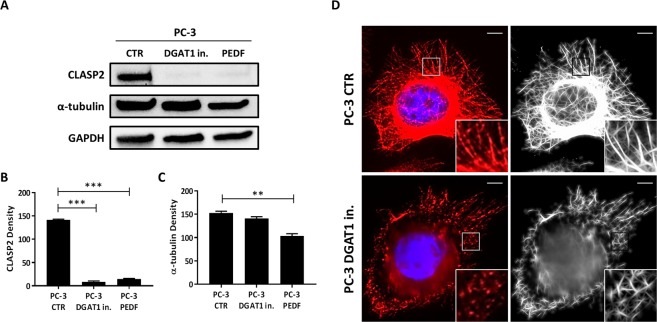
DGAT1 inhibition regulates critical MT-associated proteins and disrupts the MT network in aggressive prostate cancer cells. (**A**) CLASP2 (141 kD) and α-tubulin (55 kD) levels were analyzed by western blot in PC-3 control (CTR) and treated with 1 µM DGAT1 inhibitor or 10 nM PEDF. (**B**) CLASP2 and (**C**) α-tubulin densities were normalized by GAPDH. (**D**) PC-3 cells were treated with 1 µM DGAT1 inhibitor for 24 h, and, after fixation, the cells were stained with rabbit anti-α-tubulin antibody (red) and DAPI (blue) to visualize the MTs and nucleus, respectively. Single color (red) images of α-tubulin were processed to enhance the network of MTs using Filament Sensor software (images in black and white). Size bar: 10 µm. For the western blot analysis, each protein has been detected by sequential staining and stripping using the same membrane. Data are presented as mean ± SEM. Student’s unpaired t test. **P < 0.01, ***P < 0.001.

MTOCs represent the points of nucleation and stabilization of microtubules^26^. MTs are tubular polymers of α- and β-tubulin dimers that form part of the cytoskeleton which is important for maintaining cell structure, shape, cytoplasmic organization and assure cell division^42,43^. In cancer, some therapeutic treatments involve the use of tubulin-binding drugs which kill tumor cells by inhibiting MT dynamics, thereby altering cell division^44^. In addition to the radial centrosomal MT array, which is mainly important for the formation of mitotic spindle during cell division, MTs originating from ncMTOCs play an important role in regulating processes such as cell polarization, migration and intracellular trafficking^34^. Organelles such as the Golgi apparatus can convert to ncMTOCs through modulation of various proteins^27^. Among these, CLASP2 represents a MT plus-end stabilizing protein recruited to the trans-Golgi network which regulates Golgi-derived MT formation and orientation^34^. To determine whether inhibition of lipogenesis or activation of lipolysis could affect proteins involved in MT nucleation, PC-3 cells were treated for 24 h with a DGAT1 inhibitor and for 48 h with PEDF, and the levels of α-tubulin and CLASP2 proteins were analyzed by western blot (Fig. 4A). When compared to the baseline, both treatments markedly decreased the levels of CLASP2 protein (PC-3 DGAT1 in. vs PC-3 CTR: 8.4 ± 1.4 vs 141.2 ± 1.2; P < 0.001; PC-3 PEDF vs PC-3 CTR: 14.7 ± 0.7 vs 141.2 ± 1.2; P < 0.001) (Fig. 4B). Unlike the DGAT1 inhibitor, the treatment with PEDF was able to statistically reduce the expression of α-tubulin in PC-3 cells when compared to the control group (PC-3 PEDF vs PC-3 CTR: 103.4 ± 3.4 vs 152.7 ± 2.7; P < 0.01) (Fig. 4C). The ability of a DGAT1 inhibitor and exogenous PEDF to suppress the expression of CLASP2 protein in PCa cells suggests that the combined treatment could prove to exert a synergistic change in MT remodeling. Since the treatment with a DGAT1 inhibitor did not seem to reduce the MT density (Fig. 4A,C), we investigated if the same treatment could have an effect on overall MT structure. The morphology of the MT network was analyzed by immunofluorescence using α-tubulin antibody (Fig. 4D). When compared to PC-3 baseline, which showed intact MT network with continuous linear tubules (see magnification in upper panel, Fig. 4D) and increased MT density in the perinuclear area of the cells, the treatment with a DGAT1 inhibitor markedly affected the structure of MTs by disrupting the linear morphology of the tubules causing fragmentation (see magnification in lower panel, Fig. 4D). It also reduced the MT density especially in the perinuclear region of the cells (Fig. 4D). These data support the concept that proteins that modulate TAG metabolism are multifunctional and they can directly regulate critical MT-associated proteins and alter the morphological structure of the MTs. This previously unrecognized direct communication link between tumoral lipid balance and the integrity of the MT network could prove to be a beneficial target for therapeutic development.

### Loss of GM130 is one potential mechanism responsible for growth suppression by a DGAT1 inhibitor

**Figure 5:**
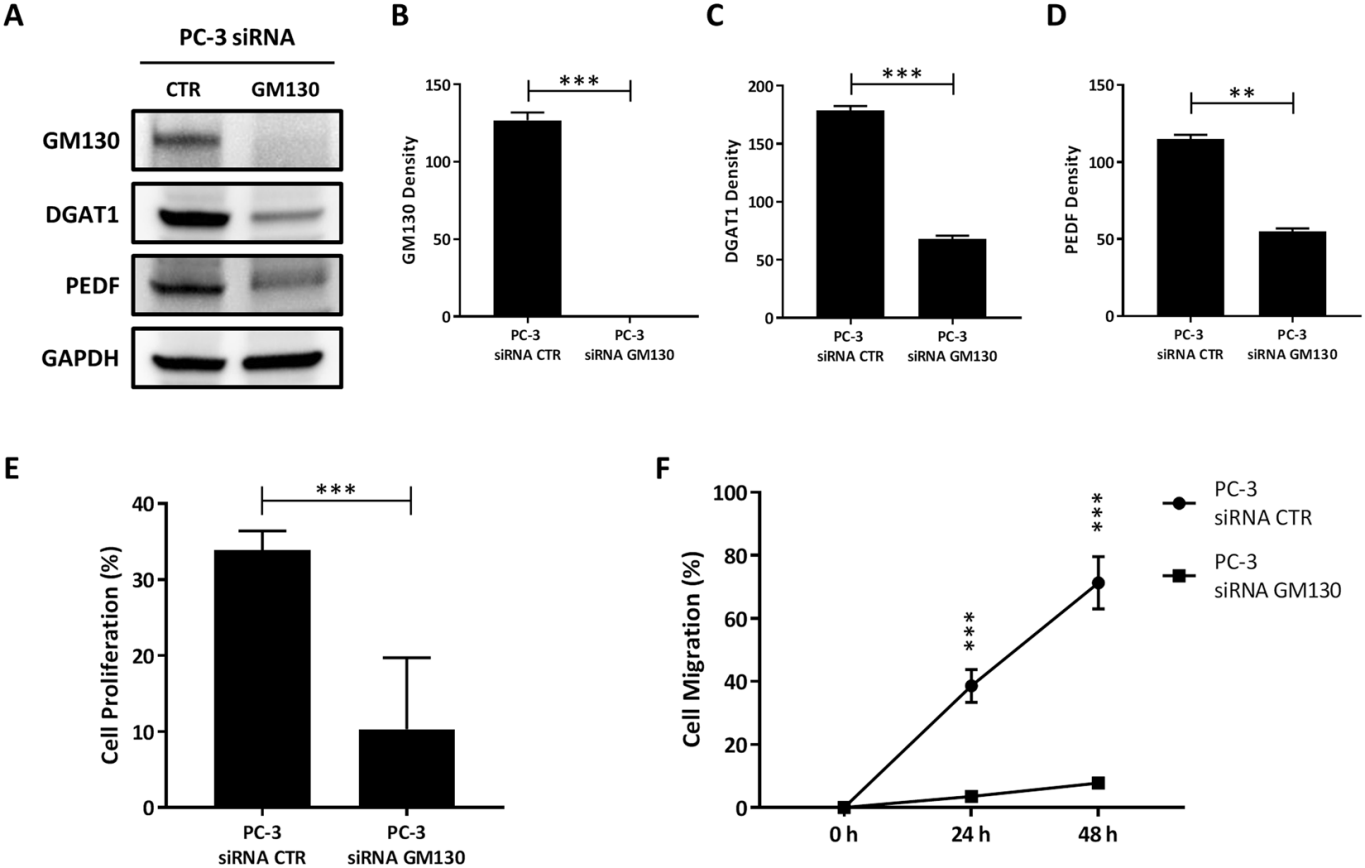
Loss of GM130 is one mechanism responsible for growth suppression induced by DGAT1 inhibitor in aggressive prostate cancer cells. (**A**) GM130 (140 kD), DGAT1 (55kD), and PEDF (50 kD) levels were analyzed by western blot in PC-3 cells transfected with siRNA targeting GM130 or with negative control siRNA. (**B**) GM130, (**C**) DGAT1, and (**D**) PEDF densities were normalized by GAPDH. (**E**) The proliferation rate of PC-3 cells transfected with siRNA targeting GM130 was evaluated by the percentage of cells positive for PCNA staining/total cells and compared to the negative control (n = 50). (**F**) Cell migration of PC-3 cells transfected with siRNA trageting GM130 was measured at the times of 24 and 48 h and compared to the negative control. For the western blot analysis, each protein has been detected by sequential staining and stripping using the same membrane. Data are presented as mean ± SEM. Student’s unpaired t test. **P < 0.01, ***P < 0.001.

Since blockade of lipogenesis by DGAT1 inhibitor strongly reduced the expression levels of the ncMTOC protein GM130 in more aggressive PCa cells, we investigated whether, in these cells, a deficiency of GM130 could have an impact on lipid metabolism and cell proliferation and migration. Depletion of GM130 was performed by small interfering RNA (siRNA) in more aggressive PC-3 cells and the levels of GM130 were evaluated by western blot and compared to the control (PC-3 siRNA GM130 vs PC-3 siRNA CTR: 1.0 ± 0.0 vs 126.8 ± 3.6; P < 0.001) (Fig. 5A,B). Surprisingly, a deficiency of GM130 strongly reduced the expression levels of DGAT1 (PC-3 siRNA GM130 vs PC-3 siRNA CTR: 68.0 ± 2.1 vs 178.7 ± 2.8; P < 0.001) and PEDF (PC-3 siRNA GM130 vs PC-3 siRNA CTR: 54.9 ± 1.5 vs 115 ± 1.9; P < 0.01) when compared to the control (Fig. 5C,D). This result suggests that GM130 is a mediator to facilitate crosstalk between lipid metabolism and ncMTOCs, supporting a novel bioactivity for GM130 in modulating the balance between lipolysis and lipogenesis.

Since inhibition of DGAT1 was able to reduce tumor cell proliferation and migration (Fig. 2A–C), we investigated whether GM130 could be one potential molecular mechanism responsible for anti-tumor activity of a DGAT1 inhibitor. Using siRNA, GM130 was knocked down by >90% in PC-3 cells, and the percentage of PCNA staining positive cells was analyzed and compared to the control (Fig. 5E). Our results showed that, compared to the control, depletion of GM130 reduced the proliferation rate nearly 70% in PC-3 cells (PC-3 siRNA GM130 vs PC-3 siRNA CTR: 10.3 ± 3.8 vs 33.9 ± 1.0; P < 0.001) (Fig. 5E). Moreover, in the same cells, loss of GM130 significantly reduced cell migration at the time of 24 h (PC-3 siRNA GM130 vs PC-3 siRNA CTR: 3.5 ± 0.3 vs 38.6 ± 5.2; P < 0.001) and 48 h (PC-3 siRNA GM130 vs PC-3 siRNA CTR: 7.8 ± 0.5 vs 71.3 ± 8.3; P < 0.001) when compared to the baseline (Fig. 5F).

### PEDF acts as an inhibitor of DGAT1 in more aggressive prostate cancer cells

**Figure 6:**
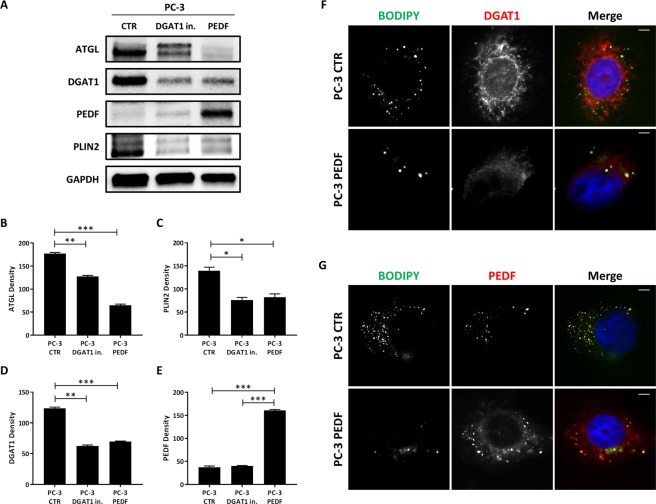
PEDF acts as an inhibitor of DGAT1 in aggressive prostate cancer cells. (**A**) ATGL (56 kD), perilipin 2 (PLIN2) (51 kD), DGAT1 (55 kD), and PEDF (50 kD) levels were analyzed by western blot in PC-3 control (CTR) and treated with 1 µM DGAT1 inhibitor or 10 nM PEDF. (**B**) ATGL, (**C**) PLIN2, (**D**) DGAT1, and (**E**) PEDF densities were normalized by GAPDH. (**F**) PC-3 cells were treated with 10 nM PEDF for 48 h, and, after fixation, the cells were stained with rabbit anti-DGAT1 antibody (red), BODIPY (green) and DAPI (blue) to localize DGAT1 protein, LDs and nucleus, respectively. Size bar: 10 µm. (**G**) PC-3 cells were treated with 10 nM PEDF for 48 h, and, after fixation, the cells were stained with rabbit anti-PEDF antibody (red), BODIPY (green) and DAPI (blue) to localize PEDF protein, LDs and nucleus, respectively. Size bar: 10 µm. For the western blot analysis, each protein has been detected by sequential staining and stripping using the same membrane. Data are presented as mean ± SEM. Student’s unpaired t test. *P < 0.05, **P < 0.01, ***P < 0.001.

To determine whether PEDF facilitates signaling events between lipolysis and lipogenesis mediators in PCa cells, the levels of TAG-related proteins were tested by western blot in PC-3 cells treated with DGAT1 inhibitor or exogenous PEDF and compared to the baseline (Fig. 6A). We postulated that ATGL-binding protein, PEDF, may act as a factor bridging the pathways regulating lipid flux. In PC-3 cells, when compared to the control, both treatments seemed to affect lipolysis by reducing the levels of ATGL (PC-3 DGAT1 vs PC-3 CTR: 127.7 ± 1.6 vs 176.9 ± 1.9; P < 0.01; PC-3 PEDF vs PC-3 CTR: 65.1 ± 1.8 vs 176.9 ± 1.9; P < 0.001) and perilipin 2 (PLIN2) (PC-3 DGAT1 vs PC-3 CTR: 75.9 ± 4.2 vs 139.5 ± 5.6; P < 0.05; PC-3 PEDF vs PC-3 CTR: 82.4 ± 5.0 vs 139.5 ± 5.6; P < 0.05) (Fig. 6B,C). Interestingly, the treatment with PEDF reduced the baseline levels of DGAT1 protein (PC-3 PEDF vs PC-3 CTR: 69.5 ± 0.7 vs 123.5 ± 1.5; P < 0.001), thus, allowing a negative feedback loop to suppress endogenous DGAT1 protein in cancer cells. To investigate the net effect on lipid content, we analyzed the lipogenesis (DGAT1)/lipolysis (ATGL or PEDF) ratio and found that DGAT1 inhibition reduced lipogenesis in the range of 29–52%. This result suggests a new role for PEDF as a potential inhibitor of the DGAT1-mediated lipogenesis in tumor cells and highlights the dynamic signaling crosstalk to consider when assessing the net lipid balance in cancer cells.

### PEDF treatment reduces LD-localized DGAT1 protein in more aggressive prostate cancer cells

To better understand the potential intracellular movement of PEDF and DGAT1, the two proteins were stained by immunofluorescence, in addition to the BODIPY-stained LDs, and their intracellular localization was detected in PC-3 cells control and cells treated with PEDF (Fig. 6F,G). At baseline, PC-3 cells showed markedly elevated DGAT1 protein levels which localized in both the cytoplasm and the surface of the LDs (Fig. 6F), while PEDF had modest expression and was located only on the LD surface (Fig. 6G). The treatment with PEDF markedly reduced the intracellular levels of DGAT1 protein, especially the protein located on LD surface (Fig. 6F), while the same treatment dispersed PEDF protein to both the cytoplasm and LD surface (Fig. 6G).

### Inhibition of DGAT1 suppresses tumor growth and invasion *in vivo*

**Figure 7:**
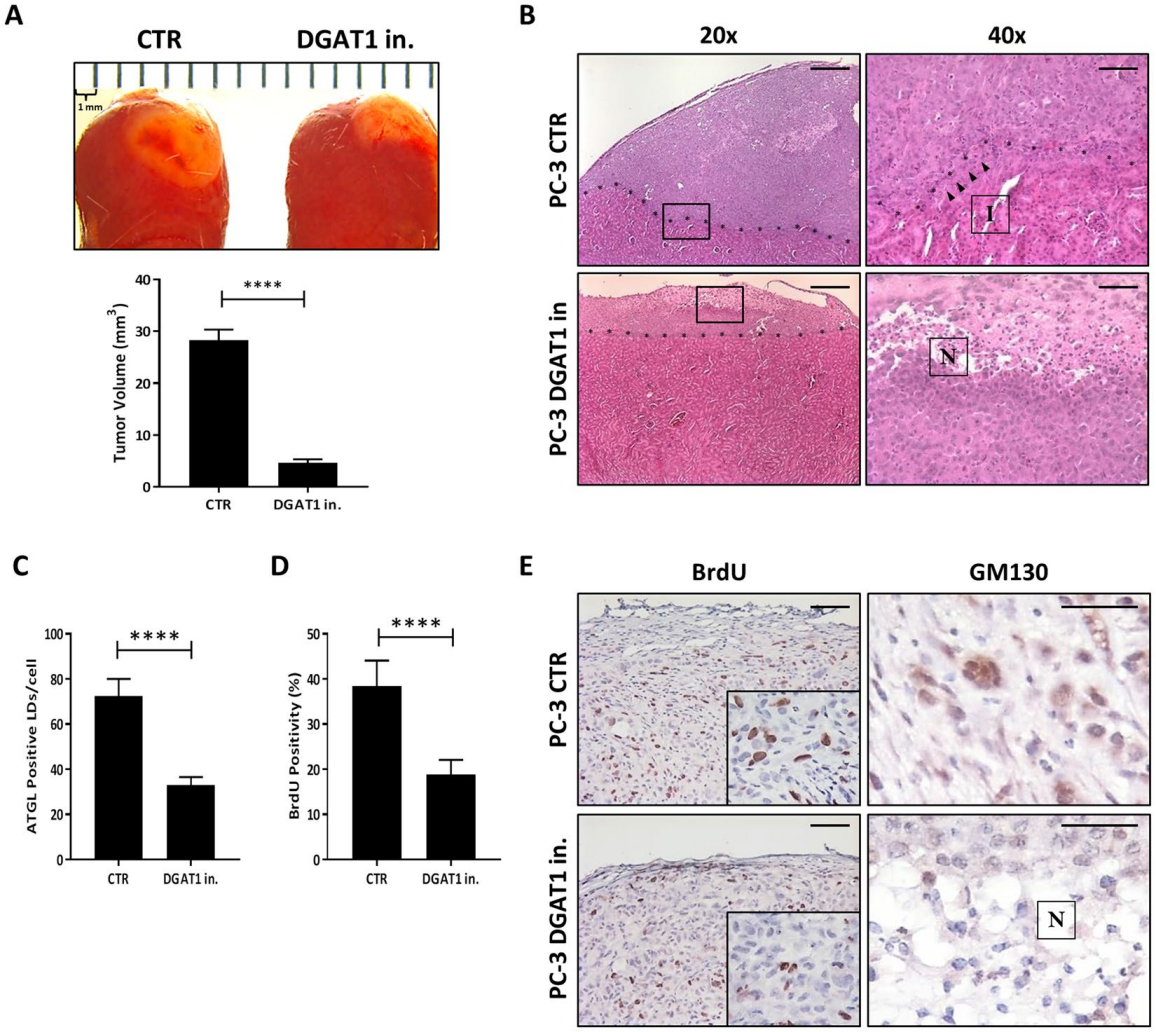
Inhibition of DGAT1 suppresses tumor growth *in vivo*. Grafts containing PC-3 cells with or without DGAT1 inhibitor were implanted under the kidney capsule of SCID mice, and, after 2 weeks, the kidneys were harvested. (**A**) Tumor volume was calculated in the treated and untreated tumors. (**B**) H&E stain was performed in kidney sections of treated or untreated tumors using 20x and 40x objectives (I: invasion; N: necrosis). Size bars: 20 μm (**C**) ATGL-positive LD density in treated vs untreated tumor tissues was analyzed (n = 50). (**D**) PC-3 cell proliferation *in vivo* was analyzed using BrdU staining (n = 50). (**E**) Immunohistochemical stains were performed for BrdU and GM130 to analyze cell proliferation and intracellular GM130 protein, respectively. Size bars: 20 μm. Data are presented as mean ± SEM. Student’s unpaired t test. ****P < 0.0001.

To assess whether the treatment with a DGAT1 inhibitor was able to suppress cancer progression *in vivo*, we implanted grafts containing PC-3 cells with or without DGAT1 inhibitor under the kidney capsule of SCID mice (mice with severe combined immunodeficiency). After 2 weeks, the kidneys were harvested and the tumor volume, histology and cancer cell proliferation were evaluated. The treatment with a DGAT1 inhibitor reduced the tumor volume nearly 84% (DGAT1 in. vs CTR: 4.6 ± 0.4 vs 28.2 ± 1.2; P < 0.0001) when compared to the untreated tumors (Fig. 7A). Sections of the untreated tumors showed a large mass of pleomorphic tumor cells elevating the renal capsule (upper 20x panel, Fig. 7B). The tumor cells demonstrated multifocal invasion (I) into the subjacent renal parenchyma (arrows in upper 40x panel, Fig. 7B). In contrast, the DGAT1 inhibitor treated tumors had less tumor volume and a large area of necrosis (N) with karyorrhectic debris (lower 40x panel, Fig. 7B). There was no evidence of invasion in the treatment group (lower 20x panel, Fig. 7B). To determine whether inhibition of DGAT1 *in vivo* altered LD density, we used a LD surface marker, ATGL, to highlight intracytoplasmic LDs. The number of LDs/cell in the treated tumors was significantly lower when compared to the untreated ones (DGAT1 in. vs CTR: 33.0 ± 1.6 vs 72.4 ± 3.4; P < 0.0001) (Fig. 7C). The *in vivo* proliferation rate of aggressive PC-3 cells was evaluated by the percentage of BrdU staining positivity (Fig. 7D,E). Compared to the control, the treatment with a DGAT1 inhibitor significantly reduced the proliferation capacity of aggressive PC-3 cells by 51% *in vivo* (DGAT1 in. vs CTR: 18.8 ± 1.0 vs 38.4 ± 1.8; P < 0.0001) (Fig. 7D). To test if the treatment with a DGAT1 inhibitor was able to reduce the levels of the ncMTOC protein GM130 also *in vivo*, immunohistochemical analysis was performed. GM130 was strongly positive in the cytoplasm of untreated tumor cells (right upper panel, Fig. 7E), while its expression was reduced in the tumors treated with a DGAT1 inhibitor (right lower panel, Fig. 7E). This result concurred with the previously shown *in vitro* western blot data (Fig. 3C,F).

## Discussion

Obesity is a significant risk factor for cancer progression and it is associated with ectopic storage of lipid in non-adipocytes throughout the body^45^. Patients with prostate cancer, hyperlipidemia and central obesity have more aggressive tumors^46^; however, how an obese microenvironment facilitates cancer cell growth is not well understood. Tumor cells undergo metabolic re-programming by increasing their rate of fatty acid synthesis to maintain adequate nutrient sources^47,48^. In this study, we postulated that the higher rate of lipid flux in prostate tumors cells is maintained, in part, by modulating the crosstalk between the key enzyme in TAG lipogenesis, DGAT1, and the lipolysis regulating proteins ATGL and PEDF. Moreover, higher levels of DGAT1 in more aggressive tumors would sustain growth and migration, whereas, blockade of DGAT1 would facilitate tumor suppressive activity.

We identified an imbalance in proteins regulating TAG metabolism in PCa cells. In normal prostate epithelial cells, PEDF was more highly expressed than ATGL and DGAT1 suggesting that this ATGL-binding protein is critical in maintaining the normal baseline lipid content. In contrast, there was a significant loss of PEDF in the prostate tumor cells and a stepwise gain in DGAT1 protein expression was observed when LNCaP was compared to the more aggressive PC-3 cell line. The imbalance in catabolic and anabolic signaling mediators appeared to trigger an increase in the lipogenesis/lipolysis ratio resulting in a net gain in stored intratumoral neutral lipid within LDs. To confirm that an increase in the DGAT1 was critical in promoting the higher lipid content and tumor cell proliferation and migration, this enzyme was blocked with a DGAT1 inhibitor. DGAT1 inhibitors are currently being tested in clinical trials as anti-obesity and insulin-sensitizing agents^22^; however, their activity as anti-tumor agents has not been investigated to date. We discovered that blockade of DGAT1 not only reduced LD density and PLIN2, but it also had potent anti-tumor activities by suppressing tumor growth both *in vitro* and *in vivo*. The DGAT1 inhibitor treated tumors had multifocal necrotic regions and less proliferative and invasive indices when compared to untreated tumors. One DGAT1-related tumor suppressive mechanism involved GM130 since targeted knockdown of this protein induced less tumor proliferation and migration *in vitro* and revealed a feedback loop linking ncMTOCs and lipogenesis. Depletion of GM130 caused a concurrent suppression in DGAT1 protein levels. These data suggested that targeting the highly expressed DGAT1 enzyme in aggressive prostate tumors could prove to be an effective therapeutic strategy to suppress tumor progression. The drug’s dual activities on both the tumor cell and the adipocyte makes it attractive since elevated body mass index is a risk modifier in patients with cancer.

DGAT1 has been found to be important in maintaining triglyceride homeostasis in other organ systems such as the heart^49^ and it appears to be a bridge to cholesterol metabolism^7^. In our recent study, we demonstrated the existence of a lipid-MTOC signaling axis in prostate stromal cells^24^; however, whether this signaling axis is active in PCa cells was not fully addressed. Although the centrosome is the most well-studied MTOC (cMTOC) and known to be amplified in tumors^50^, a significant number of non-centrosomal MTOCs have been identified originating from structures such as the Golgi apparatus^51^. Unlike cMTOCs which are involved in mostly mitotic processes, the MTs arising from ncMTOC regions tend to be asymmetric and linked to non-mitotic processes such as cell migration and intracellular trafficking^26^. This asymmetry could help explain the polarization function of ncMTOCs^33^. A group of proteins, CLASPs, have been shown to be crucial in non-centrosomal MT nucleation, especially at the trans-Golgi membrane^33^. *Cis-*Golgi-associated protein, GM130, is an important protein involved in MT nucleation at the Golgi apparatus and regulation of centrosomal structure and function during interphase^30,32^. Studies showing that MTs nucleated by the Golgi are often oriented toward the leading edge of cell and the observation that GM130 binds and activates an important cell migration protein kinase, YSK1, underscores the importance of the communication between the Golgi and MTOCs in modulating cell polarization and migration. Ninein is another critical MTOC protein which has a strong MT anchoring ability, can directly interact with ɣ-TuRC and possibly move ɣ-TuRC to the centrosome^26^. Ninein appears to also function in anchoring MTs at ncMTOCs. Several studies suggest that there is a precise coordination between the activities at cMTOCs and ncMTOCs. When ncMTOCs want to activate MT nucleation and anchoring, it requires a concurrent inactivation of these MT-related activities at the centrosomal sites^26^. Here, we provide evidence to support that a more direct signaling pathway exists between two organelles, LDs and MTOCs. The results of our study suggest that MTOCs can sense a lipid-enriched state within cancer cells. When lipogenesis is blocked through a DGAT1 inhibitor or when ATGL-binding protein, PEDF, is restored, there is a broad dampening in the expression of key MTOC regulating proteins, including CLASP2, GM130, pericentrin, γ-tubulin, and ninein. Inhibition of DGAT1 not only helped normalize amplified MTOCs, but it also induced remodeling of MTs by selectively reducing central MT density and leaving only fragmented MT remnants in the peripheral zone.

**Figure 8:**
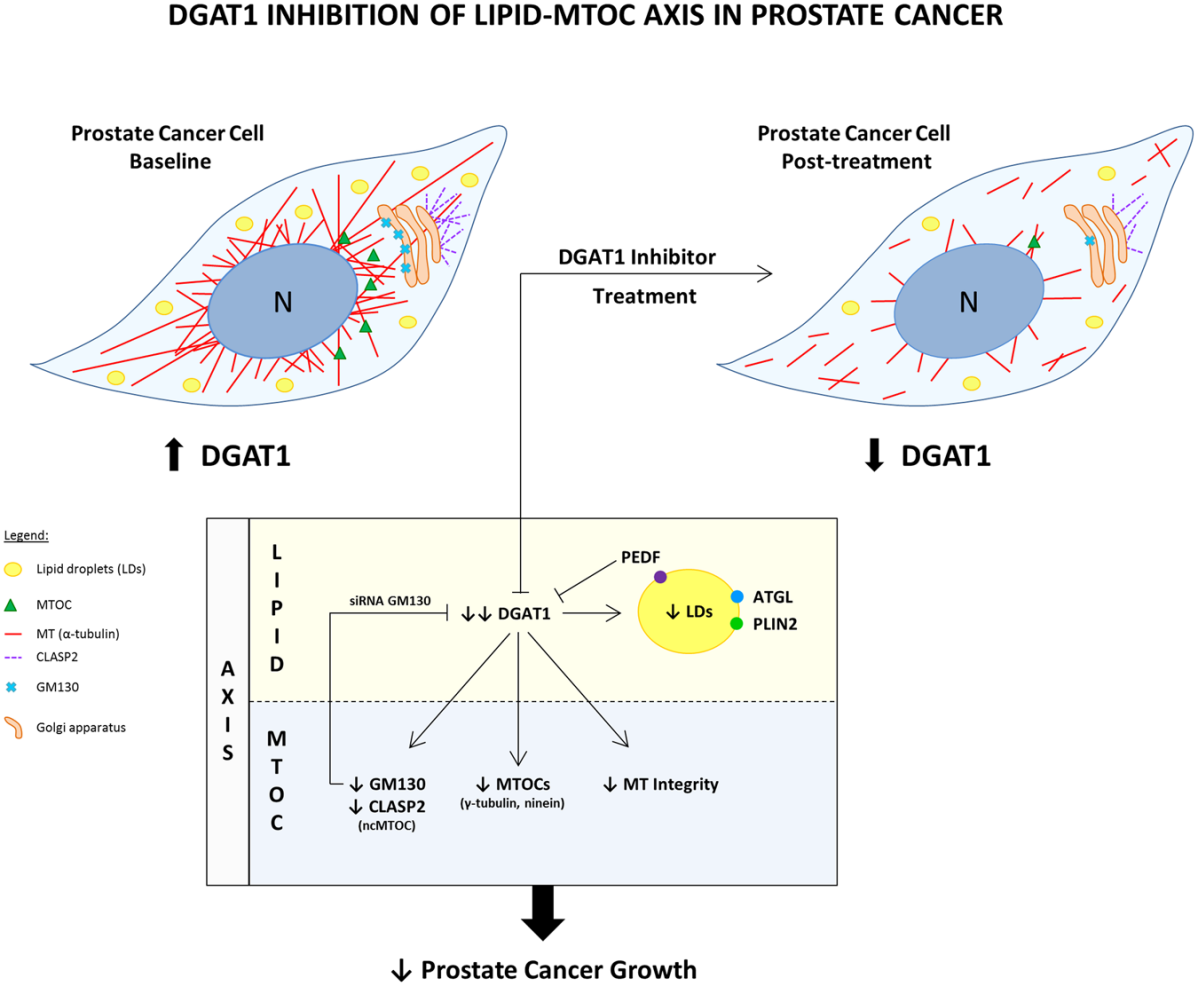
Model of DGAT1 inhibition of lipid-MTOC axis in prostate cancer. This is a model of lipid-MTOC crosstalk in prostate cancer. At baseline, PCa cells are lipid-rich with MTOC amplification and a complex and dense MT network. At the site of the Golgi apparatus, GM130 and CLASP2-coated MTs are strongly expressed. PC-3 cells express high levels of DGAT1 and low levels of PEDF protein. After treatment with a DGAT1 inhibitor, the LD density is reduced as well as the number of MTOCs. The expression levels of proteins regulating ncMTOCs (GM130 and CLASP2) and MTOCs in general (γ-tubulin and ninein) were significantly decreased. Structural changes in the MT network post-treatment revealed fragmentation and loss of many linear MTs. Blockade of the lipid-MTOC axis via DGAT1 inhibitor resulted in suppression of prostate cancer growth and invasion both *in vitro* and *in vivo*.

Precise coordination between mediators of lipogenesis and lipolysis help maintain the net amount of stored intracellular lipid in cells. Dysregulation of this balance can be due to a gain of a lipogenesis enzyme such as DGAT1, loss of lipolysis factors, or both. We found that the neutral lipid accumulation observed in PCa cells was associated with an increase in DGAT1 expression and a decrease in PEDF supporting the concept that defects are present in the TME involving both lipogenesis and lipolysis. A new finding that emerged from this study is that ATGL-binding protein, PEDF, appears to be a bridging protein between these two sides of TAG metabolism. In hepatocytes and other cell types, the interaction of PEDF with ATGL, was found to be critical in promoting lipolysis^47,48^ and, here, we observed that PEDF was able to significantly suppress DGAT1 protein levels, thus, creating a negative feedback loop between lipolysis and lipogenesis. These data suggest that the loss of PEDF in cancer cells can have more profound effects in lipid flux due to a deficiency in the dual functions of PEDF as a pro-lipolytic protein as well as an inhibitor of DGAT1-mediated lipogenesis. In summary, blockade of the DGAT1 enzyme, through treatment with a selective inhibitor or by PEDF restoration, has promising tumor suppressive functions in PCa by regulating lipid metabolism and one potential mechanism involves decreasing ncMTOC regulator GM130 (see the model Fig. 8).

## Materials and Methods

### Cell Culture and Reagents

In this study the experiments were performed using one primary human normal prostate epithelia cell line (NHPrE) and two human prostate cancer cells, LNCaP (less aggressive) and PC-3 (more aggressive), which were purchased from ATCC (Manassas, VA). LNCaP cell line was cultured at 37 °C in 5% CO_2_ in RPMI medium containing 10% fetal bovine serum (FBS) and 1% penicillin/streptomycin; PC-3 cell line was cultured in DMEM medium containing again 10% FBS and 1% penicillin/streptomycin; and NHPrE was cultured in DMEM medium containing 5% FBS, 1% insulin-transferrin-sele- nium-X, (ITS), 0.4% bovine pituitary extract (BPE), 10 ng/ml epidermal growth factor (EGF), and 1% penicillin/streptomycin as previously described^52^. After reaching 80% to 90% confluence, the cells were harvested with 0.25% trypsin - EDTA and passaged at a ratio of 1:2. After seeding overnight, the cells were exposed to two treatments: 1 µM DGAT1 inhibitor (Cayman Chemical, Ann Arbor, MI) for 24 h and 10 nM full length pigment epithelium-derived factor (PEDF - BioProducts MD, Middletown MD) for 48 h.

### Oil-Red-O Staining

LNCaP and PC-3 cells were grown on glass coverslips coated with poly-L-lysine and cultured and treated as described above. The cells were then washed three times with PBS, fixed in 10% formalin (20 min. at room temperature) and stained using Oil-Red-O (Oil Red O Stain, Propylene Glycol – Newcomer Supply, Middleton, WI). The coverslips were mounted on glass slides and sealed with permaslip. Pictures were taken of representative fields for each treatment using a 100x objective in order to count the single intracellular LDs.

### Western Blotting

NHPrE, LNCaP, and PC-3 cells cultured on dishes were treated as described above and, after 24–48 h, they were washed with PBS, scraped and lysed in M-PER + protease inhibitor buffer. Cell lysates were centrifuged at 12000 rpm for 20 min. at 4 °C, and then the protein concentration was determined using Pierce 660 nm Protein Assay Reagent and comparing it with that of a standard. Proteins separated by precast gels were transferred onto 0.2 um polyvinylidene difluoride (PVDF) membranes (Bio-Rad, Des Plaines, IL), blocked in 5% bovine serum albumin (BSA) in PBS-T, and incubated with primary and secondary antibodies. PEDF (1:1000 - Cat. No. AB-PEDF4 - BioProducts MD, Middletown, MD), ATGL (1:200 – Cat. No. 10006409 - Cayman Chemical, Ann Arbor, MI), DGAT1 (1:10000 – Cat. No. ab181180 - Abcam, Cambridge, United Kingdom), perilipin 2 (PLIN2) (2 μg/ml – Cat. No. LS-B3121 – Lifespan Biosciences, Seattle, WA), γ-tubulin (1 μg/ml – Cat. No. ab27074 - Abcam, Cambridge, United Kingdom), centrin 1 (CETN1) (1:2000 – Cat. No. ab11257 - Abcam, Cambridge, United Kingdom), ninein (1:5000 – Cat. No. NB100-74631 – Novus Biologicals, Littleton, CO), GM130 (1:5000 – Cat. No. ab52649 - Abcam, Cambridge, United Kingdom), CLASP2 (1:1000 – Cat. No. ab95373 - Abcam, Cambridge, United Kingdom), and α-tubulin (1 μg/ml – Cat. No. ab15246 - Abcam, Cambridge, United Kingdom) antibodies were used. GAPDH (1:1000 – Cat. No. 2118 - Cell Signaling Technology, Danvers, MA) antibody was used as normalizer. The membrane was then incubated at room temperature with enhanced chemiluminescence (ECL) detection reagents (Bio-Rad, Des Plaines, IL), and the bands were visualized by chemiluminescence using ChemiDoc Touch Imaging System (Bio-Rad, Des Plaines, IL).

### Proliferation Assay

The proliferation analysis was performed using two different methods: the proliferating cell nuclear antigen (PCNA) staining for PCa cells and the Vibrant MTT Cell Proliferation Assay Kit (Molecular Probes, Eugene, OR) for pancreatic and liver cancer cells. LNCaP and PC-3 cells were grown and treated with 1 μM DGAT1 inhibitor for 24 h on glass coverslips. After the treatments, the cells were washed three times with PBS, fixed in 4% paraformaldehyde for 20 min., and stained with PCNA antibody (1:40 – Cat. No. M0879 - Dako, Denmark). After the staining, the coverslips were mounted on glass slides and the percentage of positive cells in the DNA synthesis phase was determined. A minimum of 50 cells were counted in each group. Regarding PANC-1 and Hepa-1c1c7 cell lines, a total of 10^4^ cells per well were seeded into 96-well culture plates and treated for 24 h with 1 μM DGAT1 inhibitor. After 24 h, the medium was replaced with 100 µl of fresh medium and 10 µl of 12 mM MTT was added to each well. After an incubation of 4 h at 37 °C, 100 µl of SDS-HCL solution was added to solubilize the formazan product for an incubation of 4 h at 37 °C. The optical density was then determined using a spectrophotometer at a wavelength of 570 nm.

### Migration Assay

LNCaP and PC-3 cells were plated in glass dishes and once confluent they were treated with 1 µM DGAT1 inhibitor. The cell surface area was then scratched with a 200 μl pipette tip, and after washing with PBS three times, the dishes were incubated with culture media in the presence or absence of DGAT1 inhibitor. The recovery capacity of the cells migrating into the scratched area was measured at the times of 12 and 36 h, and compared with that of the control (time 0).

### Matrigel Invasion Assay

The invasion rate of LNCaP and PC-3 cells was evaluated using Matrigel. 50 μl of diluted Matrigel was pipetted into the upper chamber of Transwell Cell Inserts (0.8 μm pore size; Corning, Corning NY) contained in 24-well plates. The Matrigel was incubated at 37°C, 5% CO_2_ for at least 30 min. prior to addition of cells to the chamber. The cell suspensions containing 2 × 10^5^ cells/ml in serum-free medium were prepared, of which 250 μl were transferred into the upper chamber. 500 μl of medium containing 10% FBS with or without 1 μM DGAT1 inhibitor treatment was added to the bottom well of the chamber. After incubation at 37°C, 5% CO_2_ for 24 h, non-invading cells as well as the Matrigel from the interior of the inserts were gently removed using a cotton-tipped swab. The inserts were fixed in 4% paraformaldehyde for 20 min., and stained for 15 min. with 1 μg/ml Hoechst 33258. The images of invaded cells were captured using confocal microscopy (Nikon Eclipse TE 2000-U microscope equipped with NIS-Element version 4 software), and the number of invaded cells per field view was counted using Image J.

### Immunofluorescence Staining

Cells were grown and treated as described above on glass coverslips. After the treatments, the cells were washed three times with PBS, fixed in 4% paraformaldehyde for 20 min., and permeabilized with 0.1% triton X-100 for 15 min. Cells were incubated for 20 min. in 5% normal horse serum first, and then with the primary and secondary antibodies. Mouse anti-pericentrin (1 µg/ml – Cat. No. ab28144 - Abcam, Cambridge, United Kingdom) and rabbit anti-α-tubulin (1:100 – Cat. No. ab15246 - Abcam, Cambridge, United Kingdom) antibodies were used to obtain the MTOCs and MTs, respectively. Rabbit anti-DGAT1 (1:100 – Cat. No. ab181180 - Abcam, Cambridge, United Kingdom), and rabbit anti-PEDF (1:100 – Cat. No. sc-25594 – Santa Cruz Biotechnology, Dallas, TX) antibodies were used to localize the proteins inside the cells. Intracellular LDs were stained with BODIPY (Thermo Fisher Scientific, Waltham, MA) for 20 min. After the staining, the coverslips were mounted on glass slides using ProLong Gold antifade reagent with DAPI (Invitrogen, Carlsbad, CA) and sealed with permaslip. At the end, the cells were imaged using confocal microscopy. To enhance the MT networks, single color images of MTs stained for α-tubulin stain were processed with Filament Sensor software v. 0.1.7^53^.

### Silencing of GM130 by transfection of siRNA into prostate cancer cells

To reduce GM130 levels in aggressive PCa cells, we transfected PC-3 cells according to the manufacturer’s instructions (ThermoFisher Scientific, Waltham, MA) with commercial small interfering RNA (siRNA) constructs targeting GM130/GOLGA2 (Cat. No. AM16708). After 72 h of transfection, cells were harvested and cultured for 48 h in fresh medium lacking siRNA in order to perform western blot, cell proliferation and migration analyses.

### *In vivo* testing of DGAT1 inhibitor

For the *in vivo* analysis we used sub-renal capsule grafting method. A total of 1 × 10^6^ PC-3 cells were pelleted and re-suspended in 30 μl neutralized rat tail collagen prepared as described previously^54,55^. Two groups of grafts with PC-3 cells were prepared: a) Grafts with no treatment (n = 6) or b) Grafts containing DGAT1 inhibitor (33.3 μM per graft) (n = 6). Cells were then incubated in a 6-well TC-treated plate and supplemented with 2–3 ml complete media overnight. The next day grafts were surgically implanted under the kidney capsule of SCID mice. Grafts were harvested after two weeks, and BrdU was injected to the mice 30 min. prior to euthanization and harvest of the kidneys. Kidneys were removed, excised and imaged before processing for histology. Graft dimensions were measured, and the resultant tumor size was calculated using the following formula: volume = width × length × depth × (π/6). This study was conducted according to US federal and state regulations and approved by the NorthShore Institutional Animal Care and Use Committee.

### Immunohistochemical Staining

The kidneys harvested from the *in vivo* experiments were subjected to immunohistochemical (IHC) analysis and evaluated by two pathologists. Tumors were confirmed with histopathological review. Immunohistochemical stains were performed by a previously described method used routinely in our laboratory^56^. Hematoxylin and Eosin (H&E) staining was performed to assess the morphology and invasion of the tumor. GM130 antibody (1: 100 – Cat. No. ab52649 - Abcam, Cambridge, United Kingdom) was used to identify the protein inside the cells, while ATGL antibody (1:40 - Cat. No. 2138 - Cell Signaling Technology, Danvers, MA) was used as LD surface marker. Following ATGL staining, intracytoplasmic ATGL-positive LDs were counted per cell in five higher power fields (40x objective) using representative sections cut from both untreated and treated tumors. BrdU (1:100 – Cat. No. MA1-81890 - ThermoFisher Scientific, Waltham, MA) staining was used to analyze the proliferation rate of tumor cells *in vivo*. A minimum of 50 cells were counted per tumor section in each group.

### Statistical Analysis

To determine differences between groups we used Student’s *t*-test, where the differences were considered statistically significant at a P value < 0.05. This analysis was performed using GraphPad Prism, version 7.03. ImageJ software was used to analyze LD number, invaded cells, and to quantify the protein levels by densitometry.

## Supplementary information

Figure S1

## Data Availability

All data generated and analyzed during this study are included in this published article.
