# Supplementary material for: DGAT1 Inhibitor Suppresses Prostate Tumor Growth and Migration by Regulating Intracellular Lipids and Non-Centrosomal MTOC Protein GM130
Source: Sci Rep. 2019 Feb 28;9:3035. doi: 10.1038/s41598-019-39537-z (PMC6395665; doi:10.1038/s41598-019-39537-z)
Supplement: Supplementary file 1 — Figure S1 [file 41598_2019_39537_MOESM1_ESM.pdf]

DGAT1 Inhibitor Suppresses Prostate Tumor Growth and Migration by Regulating Intracellular Lipids and Non-Centrosomal MTOC Protein GM130

Francesca Nardi[1], Omar E. Franco[1], Philip Fitchev[1], Alejandro Morales[1], Renee E. Vickman[1], Simon W. Hayward[1], *Susan E. Crawford[1]

[1] Department of Surgery, NorthShore University Research Institute, Affiliate of University of Chicago Pritzker School of Medicine, Evanston, IL 60201, United States.

*Corresponding Author:

Dr. Susan Crawford

crawford1@uchicago.edu

Figure S1

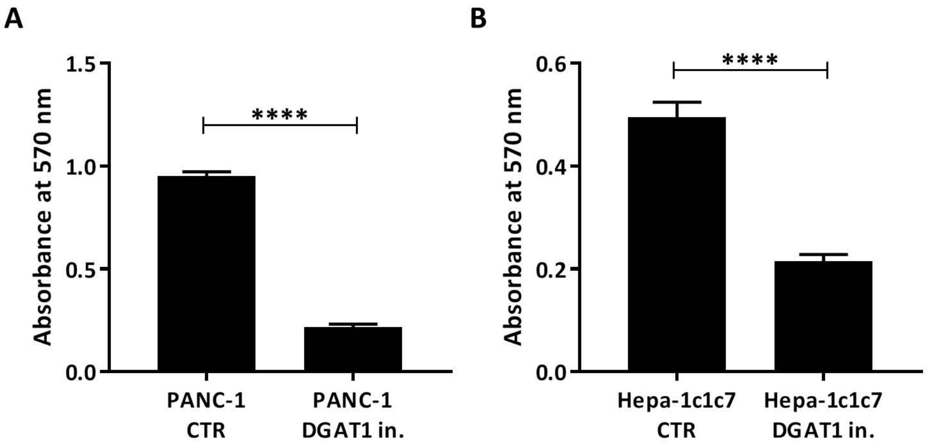
A
B
Absorbance at 570 nm
1.5
1.0
0.5
0.0
****
PANC-1 CTR
PANC-1 DGAT1 in.
0.6
0.4
0.2
0.0
****
Hepa-1c1c7 CTR
Hepa-1c1c7 DGAT1 in.

Fig. S1. DGAT1 inhibitor reduces cell proliferation in pancreatic and liver cancer cells. (A) PANC-1 and (B) Hepa-1c1c7 cells were treated with 1 μM DGAT1 inhibitor for 24 h and the proliferation rate was analyzed by the MTT Proliferation Assay. n=50 cells. Data are presented as mean ± SEM. Student's unpaired t test. ****P<0.001.
